# Siderophore biosynthesis genes of *Rhizobium* sp. isolated from *Cicer arietinum* L.

**DOI:** 10.1007/s13205-013-0164-y

**Published:** 2013-09-05

**Authors:** Bejoysekhar Datta, Pran K. Chakrabartty

**Affiliations:** 1 Department of Botany, University of Kalyani, Nadia, Kalyani, West Bengal 741 235 India; 2 Acharya J.C. Bose Biotechnology Innovation Centre, Madhyamgram Experimental Farm, Madhyamgram, Kolkata, West Bengal 700 129 India

**Keywords:** Chickpea, Nodulation, *Rhizobium*, Siderophore biosynthesis genes

## Abstract

*Rhizobium* BICC 651, a fast-growing strain isolated from root nodule of chickpea (*Cicer arietinum* L.), produced a catechol siderophore to acquire iron under iron poor condition. A Tn5-induced mutant (B153) of the strain, BICC 651 impaired in siderophore biosynthesis was isolated and characterized. The mutant failed to grow on medium supplemented with iron chelator and grew less efficiently in deferrated broth indicating its higher iron requirement. The mutant produced less number of nodules than its parent strain. The Tn5 insertion in the mutant strain, B153, was located on a 2.8 kb *Sal*I fragment of the chromosomal DNA. DNA sequence analysis revealed that the Tn5-adjoining genomic DNA region contained a coding sequence homologous to *agbB* gene of *Agrobacterium tumefaciens* MAFF301001. About 5 kb genomic DNA region of the strain BICC 651 was amplified using the primers designed from DNA sequence of agrobactin biosynthesis genes of *A. tumefaciens* MAFF 301001 found in the database. From the PCR product of the strain BICC 651, a 4,921 bp DNA fragment was identified which contained four open reading frames. These genes were designated as *sid*, after siderophore. The genes were identified to be located in the order of *sid*C, *sid*E, *sid*B, and *sid*A. Narrow intergenic spaces between the genes indicated that they constitute an operon. Phylogenetic analyses of deduced *sid* gene products suggested their sequence similarity with the sequences of the enzymes involved in biosynthesis of catechol siderophore in other bacteria.

## Introduction

In iron deficient environment, bacteria meet their iron requirement by producing low-molecular-mass iron-chelating compounds called siderophores. The compounds bind available Fe^3+^ with high affinity to form complexes which are internalized by the cells with the help of cognate membrane proteins. The structures of these compounds are quite variable. Many of them are catecholate in nature. Catecholate siderophores were isolated from a variety of bacterial species including *Escherichia coli* which produces enterobactin, the prototype of catecholate siderophore. Enterobactin is biosynthesized from 2,3-dihydroxybenzoic acid (DHBA) which, in turn is synthesized from chorismate via the consecutive actions of three enzymes, viz., isochorismate synthase (EntC), isochorismatase (EntB) and 2,3-dihydro-2,3-dihydroxybenzoate dehydrogenase (EntA) (Earhart 1996). Although, the enzymes involved in the pathway of DHBA biosynthesis are common to all catechol siderophore-producing organisms, the organization of the biosynthetic genes within the operon and the sequences of the genes are different.

The members of the genus *Rhizobium* are soil bacteria that enter into symbiotic relationship with plant hosts. This relationship is iron dependent, since iron is required for nodule formation, and synthesis of leghemoglobin, nitrogenase complex, ferredoxin and other electron transport proteins to energise the nitrogenase system during symbiosis (Guerinot 1991). Because a part of life cycle of *Rhizobium* requires invasion, growth and differentiation within its host plant tissue, the role of siderophore for iron acquisition is important for the plant–microbe interaction. Improved iron scavenging properties of *Bradyrhizobium* sp. and *Sinorhizobium meliloti* 1021 positively correlate with rhizospheric growth and nodulation effectiveness in peanut and in alfalfa (O’Hara et al. 1988; Gill et al. 1991). Moreover, siderophore-producing ability helps in the sustenance of rhizobia in iron-deficient soils (Lesueur et al. 1995). Benson et al. (2005) reported that the gene *fegA* required for utilization of ferrichrome of *B. japonicum* 61A152 was also required for symbiosis. When the gene with its native, promoter was cloned and transferred to *Mesorhizobium* sp. GN25, nodulating peanut (Joshi et al. 2008) and *Rhizobium* sp. ST1, nodulating pigeonpea (Joshi et al. 2009), the transconjugants became symbiotically more efficient indicating importance of iron uptake protein in rhizobium-legume symbiosis.

Siderophore biosynthesis genes were studied in *Rhizobium leguminosarum* bv. *viciae* (Carter et al. 2002) and in *Sinorhizobium meliloti* 1021 (Lynch et al. 2001). In both the systems, siderophore biosynthesis genes were located on plasmids and were clustered close to the genes encoding their cognate membrane proteins. Siderophores produced by these strains were not of catechol type. Catecholate siderophores were isolated from *R. leguminosarum* bv. *trifolii* (Skorupska et al. 1989), *R. ciceri* (Roy et al. 1994; Berraho et al. 1997), *Bradyrhizobium* (cowpea) (Modi et al. 1985), *Bradyrhizobium* (peanut) (Nambiar and Sivaramakrishnan 1987), but the biosynthetic genes have not been investigated yet.

A fast-growing *Rhizobium* strain BICC 651 was isolated from a nodule produced on root of a chickpea (*Cicer arietinum* L.) plant grown in the Experimental Farm of Bose Institute at Madhyamgram, West Bengal, India. The strain produced a catechol siderophore and its cognate membrane receptor in response to iron deficiency (Roy et al. 1994). Structurally, the siderophore produced by *Rhizobium* BICC 651 contains 2,3-dihydroxybenzoic acid as core compound with two moles of threonine as the ligand. This suggested that the strain BICC 651 is likely to possess enzymes for siderophore biosynthesis similar to those of other catechol siderophore-producing organisms and the biosynthetic mechanism of the siderophore could be similar to that in other catechol siderophore-producing organisms. In the present study, transposon Tn5-*mob* insertional mutagenesis of the *Rhizobium* strain BICC 651 was performed to generate mutants impaired in siderophore production. One of the mutants was exploited to find out siderophore biosynthetic genes of the *Rhizobium* strain BICC 651.

## Materials and methods

### Bacterial strains, plasmid and culture media

Bacterial strains and plasmid used in the study are listed in Table 1. *Escherichia coli* and *Rhizobium* strains were cultured in Luria–Bertani (LB) medium and yeast extract mannitol (YEM) medium, respectively. The complete medium described by Modi et al. (1985) [composition (g/l): K_2_HPO_4_, 0.5; MgSO_4_. 7H_2_O, 0.4; NaCl, 0.1; mannitol, 10; glutamine, 1; and NH_4_NO_3_, 1; pH 6.8] was used to study the growth and siderophore production of the strain BICC 651 and its mutants. For the purpose, the medium was deferrated with hydroxyquinoline (Meyer and Abdallah 1978). CAS-agar plate for detection of siderophore production by the organisms was prepared according to Schwyn and Neilands (1987) using the complete medium.

**Table 1:** Bacterial strains and plasmid used in the study

Strains and plasmid	Relevant characteristic(s)	References and/or source
*Escherichia coli* S17.1	Conjugative donor for pSUP5011	Laboratory collection
*Rhizobium* BICC 651	Wild type, Sid^+a^	Roy et al. (1994)
*Rhizobium* BICC 651R	Sid^+^, Rif^r^ (spontaneous)	This study
*Rhizobium* B153	Sid^−^:: Tn5-*mob*, Rif^r^ Neo^r^	This study
Plasmid pSUP5011	pBR325(Bam^−^)::Tn5-*mob*, Ap^r^ Neo^r^ Cm^r^	Simon (1984)

^a^Ability to produce siderophore is denoted by Sid^+^, inability is denoted by Sid^−^

### Tn5 mutagenesis

Transconjugates were generated by plate mating method (Mukhopadhyaya et al. 2000). *Escherichia coli* S17.1, harbouring the suicide plasmid pSUP5011::Tn5-*mob* (Simon 1984) was used as the donor and a rifampicin (100 μg/ml) resistant spontaneous mutant of the *Rhizobium* strain BICC 651, designated as *Rhizobium* BICC 651R was used as the recipient. Fresh cultures of both the donor and the recipient were mixed, centrifuged to collect the cells and spread on plates containing tryptone-yeast extract agar medium [composition (g/l): bactotryptone, 8; bactoyeast, 5; NaCl, 5; and agar 20; pH 6.8] (Beringer 1974). The mating mixture was incubated overnight at 30 °C. The cells were then collected, suspended in YEM broth and plated on Rhizobium medium (Himedia) containing neomycin (50 μg/ml) and rifampicin (100 μg/ml). The plates were incubated at 28 °C for the development of isolated colonies of transconjugants. The colonies were picked to prepare master plates for post mating selection. Each master plate was replica plated on CAS plate. The mutants which did not produce orange halo were selected as siderophore negative (Sid^−^) mutants.

### Bacterial growth and siderophore production

The parent strain BICC 651R, and its Sid^−^ mutant, B153, were examined for their growth on Rhizobium medium in presence of increasing concentrations of bipyridine, a synthetic iron chelator. Subsequently, the parent strain and the mutant were studied for their growth and siderophore production in deferrated complete medium. The cultures were incubated on a shaker at 28 °C and growth was measured by monitoring OD_590_ of the culture. Siderophore in the culture supernatant was assayed using the CAS reagent (Schwyn and Neilands 1987).

### Plant inoculation and nodulation assay

For nodulation study, surface-sterilized seeds of chickpea were inoculated with *Rhizobium* strains and planted in sea sand in pots. Sea sand was treated with HCl, washed with distilled water until the pH of the washing was neutral. It was then air dried and fumigated with chloroform overnight under airtight condition, autoclaved and finally kept at 200 °C in a hot air oven for 6 h. Five hundreds gram of sterile sand in an earthen pot was moistened with 100 ml of nitrogen-free plant nutrient medium for sowing the seeds. The composition of the nitrogen-free medium was (g/l): CaSO_4_. 2H_2_O, 0.34; K_2_HPO_4_, 0.17; MgSO_4_. 7H_2_O, 0.25; Fe Citrate, 0.002; KCl, 0.075; trace element solution, 0.5 ml; pH 7.2 (Norris and Date 1976). The composition of the trace element solution was (g/l): ZnSO_4_. 7H_2_O, 2.25; CuSO_4_. 5H_2_O, 1.00; MnSO_4_. 5H_2_O, 0.50; CaCl_2_. 2H_2_O, 2.00 Na_2_B_4_O_7_. 10H_2_O, 0.23; (NH_4_)_6_Mo_7_O_24_, 0.10. Surface sterilization of chickpea seeds was carried out by soaking in concentrated H_2_SO_4_ for 10 min, then washing the seeds several times with sterile distilled water. The parent strain BICC 651R and its Sid^−^ mutant B153, were grown in YEM broth till late log phase, and the harvested cells were mixed with sand-charcoal (1:3) containing 2 % aqueous sodium carboxymethyl cellulose. The mixture was used to coat the surface-sterilized seeds. The coated seeds (~10^8^ bacteria/seed) were kept in dark for overnight and the next day, five seeds were transferred to each pot containing the sterile sand soaked in nitrogen-free plant nutrient medium. A control set of seeds which did not receive any inoculum was also included in this study. The pots were kept under well-illuminated condition and watered when necessary to moisten the sand. At 1 week interval, 50 ml of 1/10th dilution of nitrogen-free plant nutrient medium was added to each pot. At 35 days of inoculation, plants were uprooted and observed for development of nodules.

### DNA preparations and Southern hybridization

Total DNA of the parent, BICC 651R and the mutant, B153, was isolated, digested with *Sal*I, electrophoresed on 1 % agarose gel according to standard protocol (Sambrook et al. 1989). DNA fragments from agarose gel were blotted on nylon membrane and hybridized with probe prepared from Tn5 fragment containing neomycin resistance (neo^r^) gene. The probe was labelled by biotinylation following the manufacturer’s instruction (NE Blot Phototope Kit, Biolabs).

### DNA ligation and inverse PCR

The *Sal*I digested DNA fragments showing positive signal following hybridization with labelled probe were eluted from the gel and allowed to self-ligate by incubating the DNA at a concentration of 0.3–0.5 μg/ml in presence of 3 U of T4 DNA ligase (Promega)/ml overnight at 4 °C (Huang et al. 2000). The ligation mixture was extracted by phenol: chloroform, the DNA was precipitated with ethanol, and dissolved in sterile distilled water to a concentration of 20 μg/ml. The inverse PCR was carried out using the self-ligated product containing a portion of the Tn5 as template and Tn5Int and NeoF as primers (Table 2). The amplifications were performed using Perkin-Elmer PCR system 2,400 in 50 μl of reaction mixture containing 1 × enzyme buffer, 1.5 mM MgCl_2_, 200 μM dNTPs (each), 500 nM of each primers and 1.5 U *Taq* DNA polymerase (Fermentas) and 50 ng of purified self-ligated product. The thermal programme used for PCR was as follows: initial denaturation for 10 min at 94 °C; 35 cycles of denaturation for 30 s at 94 °C, annealing for 30 s at 55 °C, and extension at 72 °C for 1 min, followed by a final extension at 72 °C for 10 min. The identity of the PCR product was confirmed by nested PCR and also by ascertaining the profile of bands resulting from its specific endonuclease digestion. The PCR product was purified and sequenced by ABI PRISM 377 automated DNA sequencer (Perkin-Elmer, Applied Biosystem, Inc.).

**Table 2:** Primers used in the study

Primers	Sequences (5′–3′)	Position of nucleotides	Sources
Tn5Int	CGGGAAAGGTTCCGTTCAGGACGC	21–34 [complementary] or 5775–5798	*E. coli* transposon Tn5 (accession no. U00004 L19385).
NeoF	CGCATGATTGAACAAGATGG	1548–1567	
agbCF	GACAGGATCGACGGACTGAC	1646–1665	Ferric iron uptake gene of *A. tumefaciens* MAFF 301001 (accession no. AB083344).
agbAR	GTAACGAAGGGTGAGGCAAT	6620–6639 [complementary]	

### Amplification of agb homologues from genomic DNA of BICC 651 and site of Tn5 insertion in Sid^−^ mutant

To amplify the homologues of agrobactin biosynthetic genes from the strain BICC 651, PCR amplifications were carried out using the genomic DNA of BICC 651 as template, and agbCF and agbAR as primers (Table 2). A long PCR amplification kit was used for the purpose following the manufacturer’s instruction (Genei, India). To identify the position of Tn5 insertion in the genomic DNA of the mutant, B153, two sets of PCR were carried out, one with the primer pair, agbCF and Tn5Int and another with the primer pair agbAR and Tn5Int. The reaction mixture for amplification contained 100 ng of genomic DNA as template and the thermal programme remained the same as described earlier for IPCR. To amplify larger (>2 kb) DNA fragment, extension time was increased by 1 min for each 1 kb of desired amplicon during final extension. The PCR products were analysed by agarose gel electrophoresis, purified and sequenced.

### Analysis of the Tn5 adjacent DNA sequence

Genomic DNA sequence was analyzed using BLAST version 2.2.1 of National Center for Biotechnology Information. Nucleotide BLAST and BLASTX were used to search for nucleotide sequences and derivative amino acid sequences, respectively.

### Genomic context analysis

Gene context analysis of *sidB* gene of *Rhizobium* strain BICC 651 was carried out using the GeConT programme. The program allowed comparison of adjacent genes of *sidB* and visualization of genomic context of *sidB* homologue in the genomes of other organisms. The program is available at: http://www.ibt.unam.mx/biocomputo/gecont.html (Ciria et al. 2004).

### Phylogenetic analysis

Evolutionary analyses were conducted using the software MEGA5 (Tamura et al. 2011). Multiple sequence alignment was carried out using a CLUSTALW and evolutionary history of the sequence was inferred using the neighbor-joining method (Saitou and Nei 1987). The optimal tree with the sum of branch length is equal to 4.31786458 is shown. The tree is drawn to scale, with branch lengths in the same units as those of the evolutionary distances used to infer the phylogenetic tree. The evolutionary distances are computed using the Poisson correction method (Zuckerkandl and Pauling 1965) and are in units of the number of amino acid substitutions per site.

## Results and discussion

### Transposon mutagenesis, and isolation of Sid^−^ mutants

Transposon insertion mutagenesis in a siderophore producing *Rhizobium* sp. of chickpea was carried out to select the siderophore negative mutants to identify the structural genes required for biosynthesis of siderophore in the organism. Random mutagenesis of the *Rhizobium* strain BICC 651R by mobilization of Tn5-*mob* from the suicide vector pSUP5011 to the recipient cells occurred at a frequency of 10^−5^ per donor as revealed by the number of neomycin-resistant transconjugants. This is about 1,000 times greater than the rate of spontaneous resistance of the recipients to neomycin (10^−8^). Similar transposition frequency had also been reported for other bacteria (Mukhopadhyaya et al. 2000; Manjanatha et al. 1992). Upon screening of the 1,000 of transconjugants, five Sid^−^ mutants were isolated. Among these, one mutant (B153) was characterized in the present study.

### Study of iron requirement

**Fig. 1:**
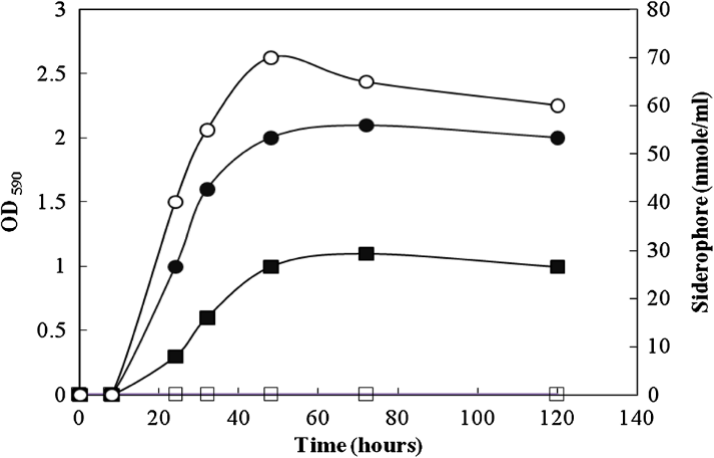
Growth (*filled circle*, *filled box*) and siderophore production (*open circle*, *open box*) of the parent strain BICC 651R and its Sid^−^ mutant B153, respectively, in the deferrated complete medium

The amount of iron required for optimal growth of many Gram-negative bacteria ranged from 0.36 to 1.8 μM (Lankford 1973). The strain BICC 651R was able to grow on Rhizobium medium containing bipyridine up to a concentration of 400 μM but the Sid^−^ mutant, B153, could not grow even in the presence of 50 μM of bipyridine (Table 3). When the organisms were grown in the complete medium deferrated with hydroxyquinoline, the highest level of growth of the Sid^−^ mutant B153 (OD_590_ = 1) was almost half as that of the parent strain (OD_590_ > 2). These observations suggested that because of impaired biosynthesis of siderophore, the mutant required more iron for its growth than the parent strain. Siderophore was not detected in the culture filtrate of the mutant at all stages of its growth, whereas the parent strain was observed to produce as much as 70 nmole of siderophore per ml of culture filtrate during late log phase of its growth (at 48 h) in the deferrated complete medium (Fig. 1).

**Table 3:** Growth of bacterial strains in presence of increasing concentrations of bipyridine

Concentration of bipyridine (μM)	BICC 651R	B153
0	+	+
50	+	–
100	+	–
200	+	–
300	+	–
400	+	–
500	–	–

The organisms were streaked on bipyridine containing Rhizobium medium and growth was scored after three days of incubation; ‘+’ indicates growth, ‘−’ absence of growth

### Plant nodulation study

Table 4 presents the data of symbiotic performance of the siderophore non producing mutant B153 and its parent strain BICC 651R. The control plants receiving no inoculum produced no nodules. The plants inoculated with B153 produced less number of nodules than those inoculated with the parent strain BICC 651R. The wet weight of nodules produced by the mutant B153 was almost half as compared to those of its parents. Gill et al. (1991) reported that mutants of *Sinorhizobium meliloti* which were unable to produce siderophore were able to nodulate the plants but the efficiency of the nodules in nitrogen fixation was less as compared to wild type incited nodules indicating the importance of iron in symbiotic N_2_ fixation.

**Table 4:** Symbiotic performance of *Rhizobium* BICC 651R and its siderophore non-producing mutant

Strains	No. of nodules per plant	Wet weight of nodules (mg) per plant	Root length (mm)	Shoot length (mm)
BICC 651R	20	872.5	100	230
B153 (Sid^−^)	14	417.2	100	200
Control	Nil	Nil	80	200

Measurements were made on the 35th day of sowing

### Identification of the DNA fragments containing Tn5

**Fig. 2:**
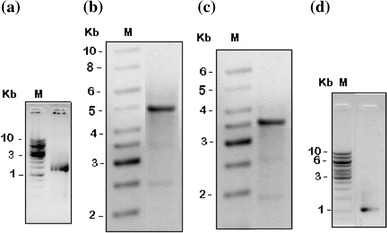
Ethidium bromide stained agarose gel showing amplified DNA fragments. **a** 1.3 kb fragment containing part of Tn5 and part of *sidB* gene of the mutant, B153. **b** 5 kb fragment containing four *sid* genes of *Rhizobium* BICC 651. **c** 3.5 kb fragment containing whole of the *sidC, sidE* genes, and part of the *sidB* gene of B153. **d** 1.5 kb fragment containing part of the *sidB* and whole of the *sidA* gene of B153

*Sal*I cuts the 7.7 kb Tn5-*mob* DNA into two parts: a 2.7 kb part with neo^r^ gene and a 5 kb part containing the 1.9 kb *mob* region. When the Tn5 probe prepared from Tn5 fragment containing neo^r^ gene was allowed to hybridize with *Sal*I restricted genomic DNAs of BICC 651R and its Sid^−^ mutant, B153, it showed positive signal only as a single band of 2.8 kb genomic DNA of the mutant indicating insertion of a single copy of Tn5 in the genome of the mutant and no hybridization of the probe was detected with the genomic DNA of the parent, BICC 651R. In the mutant, the 2.8 kb *Sal*I restricted genomic DNA fragment contained the 2.7 kb Tn5 fragment adjoining nearly a 0.1 kb genomic DNA fragment. The 2.8 kb *Sal*I fragment was used to self-ligate more easily than using a larger (>7.7 kb) *Eco*RI restricted fragment. The Tn5 adjoining genomic DNA fragments were amplified by inverse PCR using the Tn5Int and NeoF primer pair (Table 2). A 1.3 kb amplicon containing an almost 0.1 kb genomic DNA was obtained (Fig. 2a). The genomic DNA fragment was sequenced and the sequence showed 94 % identity with the *agbB* gene sequence of *Agrobacterium tumefaciens* MAFF301001 (GenBank accession no. AB083344). The *agbB* gene product, isochorismatase, catalyzes the synthesis of 2,3-dihydro 2,3-dihydroxybenzoic acid from isochorismate (Sonoda et al. 2002). Thus, in the mutant, B153, Tn5 interrupted one of the DHBA biosynthetic genes and resulted in impaired synthesis of siderophore.

### Nucleotide sequence analysis of the siderophore biosynthesis region

**Fig. 3:**
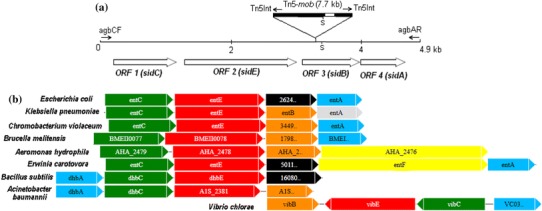
Genetic organization and genomic context of the siderophore biosynthesis DNA region of *Rhizobium* mutant, B153. **a** The physical map showing organization of *sid* genes, transcriptional directions of four ORFs, site of Tn5 insertion, *Sal*I (S) restriction sites, alignment site of the primers (agbCF, agbAR and Tn5Int) and hybridization site of the Tn5 probe (marked as *white*). **b** Genomic context and organization conservation of the *sidB* gene region in the genome of *Rhizobium* BICC 651 in comparison to the genomes of related catechol-producing organisms. *Arrows* represent genes with their relative orientation in the genomes. Genes are *coloured* according to their functional categories and labelled according to their original gene annotation in the database. *Orange/black* (isochorismatase); *red* (2,3-dihydroxybenzoate-AMP-ligase); *green* (isochorismate synthase); *sky blue/grey* (2,3-dihydro-2,3-dihydroxybenzoate) and *yellow* (entF)

Using the agbCF and agbAR primer pair, a 5 kb DNA fragment was amplified from the genomic DNA of BICC 651 (Fig. 2b). From the fragment, 4,921 bp was sequenced using appropriate primers and the DNA sequence was submitted to GenBank under accession no. GU251056. Upon nucleotide BLAST search, the 4,921 bp DNA region of BICC 651 was found to match (90 % identity) with the agrobactin biosynthetic (*agb*) genes of *A. tumefaciens* MAFF301001 (accession no. AB083344). Nucleotide sequence analysis of the 4,921 bp DNA region of BICC 651 revealed the presence of four open reading frames (ORFs) (Fig. 3a). *ORF1* (*sidC*) was 1,029 bp long (positions 213–1,241), potentially coding for a 342-amino-acid-residue-long protein. *ORF2* (*sidE*), identified between positions 1,406–3,034, was 1,629 bp long and coded for a putative protein of 542 amino acid residues. At 97 bp, downstream from the TGA stop codon of *ORF2* was found the *ORF3* (*sidB*), 870 bp long, potentially coding for a 289-amino-acid-residue protein (positions 3,131–4,000). Finally, at 14 bp from the TGA stop codon of *ORF3*, the ATG start codon of *ORF4* (*sidA*) (positions 4,014–4,772) was located. It was only 759 bp long, with a predicted translation product of 252-amino acid residues. The four ORFs identified in the 5 kb region were closely connected with narrow intergenic spaces indicating their polycistronic organization within an operon. In the sequence of 4,921 bp, a probable ribosome-binding site (AGGAGG) was identified six bp upstream of the ATG start codon of *sidC* of BICC 651 using Promoter prediction search tool (www.softberry.com). A presumable iron box (ACAAAACATGATTAGC) was also identified 56 bp upstream of the *sidC* start codon similar to the one found in *A. tumefaciens* MAFF301001 (accession no. AB083344) and in *Pseudomonas fluorescens* (accession no. Y09356). Presence of the potential Fur box sequence indicated iron regulation of the functions of *sid* genes in the strain BICC 651.

### Site of Tn5 insertion

From the genomic DNA of the mutant, B153, a 3.5 kb product was obtained using agbCF and Tn5Int primers (Fig. 2c) and a 1.5 kb product was amplified using agbAR and Tn5Int primers (Fig. 2d). Matching the sequences, it was found that Tn5 was inserted next to the 210th nucleotide position from the start codon of the *sidB* gene in the mutant B153 (Fig. 3a).

### Comparison of genetic organization of *sid* genes of BICC 651 with other species

The genomic context of *sid* DNA region in *Rhizobium* BICC 651, when compared to those in other catechol siderophore-producing members revealed that the organization of the region was not conserved; relative orientation and arrangement of the genes in the region of the genomes of different members varied considerably (Fig. 3b). Genetic organization of *sidC*, *sidE*, *sidB*, and *sidA* of *Rhizobium* BICC 651 was similar to that of *entCEBA* in *E. coli* for enterobactin biosynthesis (Crosa and Walsh 2002), in *A. tumefaciens* MAFF301001 for agrobactin (Sonoda et al. 2002), and siderophore biosynthetic genes in *Brucella melitensis*, *Chromobacterium violaceum*, and *Klebsiella pneumoniae*. In *Aeromonas hydrophila*, *entA* was missing immediately after *entB,* whereas in *Erwinia carotovora*, *entF* was present in between *entB* and *entA*. The four biosynthesis genes were arranged as *dhbACEB* cluster in *Bacillus subtilis* (Rowland et al. 1996) as well as in *Acinetobactor baumannii* (Dorsey et al. 2003). In *Vibrio cholerae* for vibriobactin biosynthesis, these were organised as *vibACEB* cluster in which *vibC* and *vibE* were co-transcribed, while *vibA* and *vibB* belonged to two independent transcriptional units (Wyckoff et al. 1997). Since organization of catechol siderophore genes of any *Rhizobium* spp. was not found in the database, gene context analysis was carried out from available data of catechol siderophore-producing organisms.

### Deduced functions of *sid* gene products

**Fig. 4:**
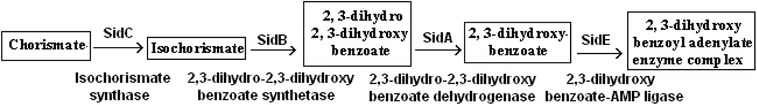
Deduced functions of the derivative amino acid sequence of *sid* gene products of *Rhizobium* BICC 651

Comparison of the derivative amino acid sequences of the genes with those of related organisms was made by BLASTX analysis (Table 5). The derivative amino acid sequence of *ORF1* (*sidC*) of *Rhizobium* BICC 651 showed highest similarity (92 %) to isochorismate synthase of *Agrobacterium tumefaciens* (accession no. BAC16757). The enzyme converts chorismate to isochorismate. The derivative amino acid sequence of *ORF2* (*sidE*) showed highest homology (89 %) to that of 2,3-dihydroxybenzoate-AMP-ligase of *A. tumefaciens* (BAC16758). The enzyme activates 2,3-dihydroxybenzoate by forming a (2,3-dihydroxybenzoyl) adenylate-enzyme complex (Rusnak et al. 1989). Consequently, the deduced gene product of *ORF3* (*sidB*) showed highest similarity (97 %) with 2,3-dihydro-2,3-dihydroxybenzoate synthetase (isochorismatase) of *A. tumefaciens* (BAC16759). The enzyme removes the enolpyruvyl side chain of isochorismate (Rusnak et al. 1990). The *ORF4* (*sidA*) translational product showed 90 % homology with 2,3-dihydro-2,3-dihydroxybenzoate dehydrogenase of *A. tumefaciens* (BAC16760). The enzyme catalyzes the NAD-coupled oxidation of 2,3-dihydro-2,3-dihydroxybenzoate (Liu et al. 1989). The deduced functions of the derivative amino acid sequence of *sid* gene products are presented in the Fig. 4.

**Table 5:** Amino acid sequence identities of products of *sid* genes of *Rhizobium* BICC 651 with related catechol-producing organisms

ORFs (accession No.)	Corresponding protein in other catechol-producing organisms (accession No.)	% identity
ORF1/*SidC*	AgbC [*Agrobacterium tumefaciens* MAFF 301001] (BAC16757)	92
(ADB12985)	Isochorismate synthase [*Rhizobium lupini* HPC(L)] (EKJ95799)	85
	Isochorismate synthase [*Brucella melitensis* 16M] (AAL53318)	60
	PmsC [*Pseudomonas fluorescens* WCS374] (CAA70528)	55
	EntC [*Chromobacterium violaceum* ATCC 12472] (AAQ59160)	44
	Isochorismate synthase [*Aeromonas hydrophila* ATCC7966] (ABK37486)	41
	EntC [*Escherichia coli* ATCC 8739] (ACA78675)	39
	EntC [*Klebsiella pneumoniae* subsp. *pneumoniae* MGH 78578] (ABR76061)	39
	VibC [*Vibrio chlorae* Lou15] (AAC45925)	34
	DhbC [*Bacillus subtilis* subsp. *subtilis* 168 (AAC44631)	33
ORF2/SidE	AgbE [*Agrobacterium tumefaciens* MAFF 301001] (BAC16758)	89
(ADB12986)	2,3-dihydroxybenzoate-AMP-ligase [*Rhizobium lupini* HPC(L)] (EKJ95800)	87
	2,3-dihydroxybenzoate-AMP-ligase [*Brucella melitensis* ATCC 23457] (ACO01901)	72
	ATP-dependent activating enzyme/PmsE [*Pseudomonas fluorescens* WCS374] (CAA70529)	64
	EntE [*Chromobacterium violaceum* ATCC 12472] (AAQ59159)	63
	2,3-dihydroxybenzoate-AMP-ligase [*Aeromonas hydrophila* ATCC7966] (ABK38474)	59
	DhbE [*Acinetobacter baumannii* ATCC 17978] (ABO12800)	59
	EntE [*Escherichia coli* ATCC 8739] (ACA78674)	55
	EntE [*Klebsiella pneumoniae* subsp. *pneumoniae* MGH 78578] (ABR76062)	55
ORF3/SidB	AgbB [*Agrobacterium tumefaciens* MAFF 301001] (BAC16759)	97
(ADB12987)	Isochorismatase [*Rhizobium lupini* HPC(L)] (EKJ95801)	95
	Isochorismatase [*Brucella melitensis* 16 M] (AAL53320)	71
	DhbB [*Acinetobacter baumannii* ATCC 17978] (ABO12799)	63
	EntB [*Chromobacterium violaceum* ATCC 12472] (AAQ59158)	62
	Isochorismatse [*Aeromonas hydrophila* ATCC7966] (ABK39064)	60
	DhbB [*Bacillus subtilis* subsp. *subtilis* 168 (AAC44633)	56
	EntB [*Klebsiella pneumoniae* subsp. *pneumoniae* MGH 78578] (ABR76063)	55
	VibB [*Vibrio chlorae* Lou15] (AAC45926)	54
	EntB [*Escherichia coli* ATCC 8739] (ACA78673)	53
ORF4/SidA	AgbA [*Agrobacterium tumefaciens* MAFF 301001] (BAC16760)	90
(ADB12988)	2,3-dihydro-2,3-dihydroxybenzoate dehydrogenase [*Rhizobium lupini* HPC(L)] (EKJ95802)	89
	2,3-dihydro-2,3-dihydroxybenzoate dehydrogenase [*Brucella melitensis* 16 M] (AAL53321)	67
	EntA [*Chromobacterium violaceum* ATCC 12472] (AAQ59157)	58
	2,3-dihydro-2,3-dihydroxybenzoate dehydrogenase [*Aeromonas hydrophila* ATCC7966] (ABK36600)	57
	EntA [*Escherichia coli* ATCC 8739] (ACA78672)	51
	DhbA [*Bacillus subtilis* subsp. *subtili*s 168] (AAC44630)	34
	VibA [*Vibrio chlorae* Lou15] (AAC45924)	34

### Phylogenetic analysis

**Fig. 5:**
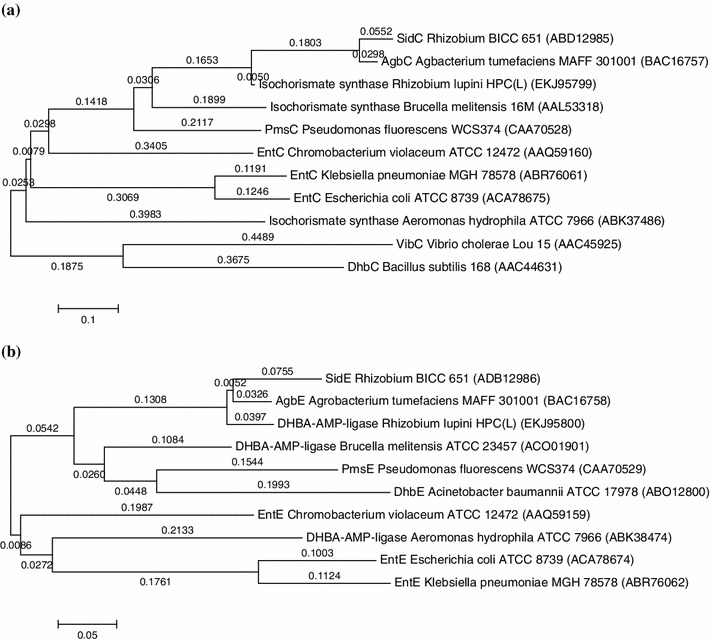

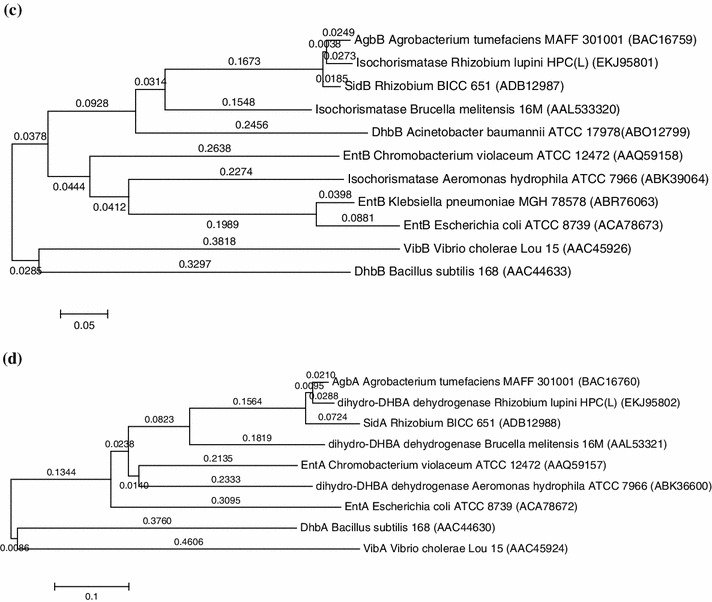
Phylogenetic trees based on comparison of the translational products of the *sid* genes. Trees were constructed by using the neighbour-joining method. Horizontal branch lengths are proportional to the estimated number of nucleotide substitutions. **a** SidC and its homologues, **b** SidE and its homologues, **c** SidB and its homologues, **d** SidA and its homologues

On the basis of the amino acid sequence identities data given in Table 5, phylogenetic tree of each of the four *sid* translational products was constructed using neighbor-joining method (Fig. 5). In all the trees, the strain BICC 651 was placed in the same evolutionary branch as of *Agrobacterium tumefaciens*. This result along with 16S Rdna sequence analysis of the strain BICC 651 (accession no. DQ839132) demonstrates that the strain BICC 651 has a chromosomal background similar to that of *A. tumefaciens*. Since the strain BICC 651 was isolated from a nodule on the root of a legume and genes responsible for symbiosis including nodulation are located on Sym plasmids of rhizobia it might be possible that the strain BICC 651 had its ancestry in *Agrobacterium* but through horizontal gene transfer acquired the Sym plasmid from some *hitherto* unknown source. A somewhat similar observation was made with a strain T1K, reported to be *Rhizobium trifolii* (*R. leguminosarum* biovar *trifolii*), which nodulated red and white clover and shared many physiological properties with *Agrobacteriumtumefaciens*. The strain was more closely related to *Agrobacterium* than to *R.trifolii* (Skotnicki and Rolfe 1978). Based on the gene sequence similarity, it is concluded that the stain BICC 651 might acquire the siderophore gene cluster from other bacteria via horizontal gene transfer during evolutionary process as suggested by Sonoda et al. (2002) who proposed that *Agrobacterium tumefaciens* MAFF301001 had obtained the *agb* gene cluster from *Brucella melitensis*, *Pseudomonas fluorescens*, *Escherichia coli* or *Salmonella typhimurium* in the same process.

Nucleotide sequence analysis revealed that the genes, *sidC*, *sidE*, *sidB*, *sidA* constitute an operon. Disruption of *sidB* by Tn5 insertion results in poor growth under iron-limiting condition, absence of siderophore production and production of less number of nodules by the Sid^−^ mutant, B153 as compared to the wild type strain. The data indicate that the siderophore biosynthetic genes are essential for growth and production of catechol siderophore of the *Rhizobium* BICC 651, which in turn influences its symbiotic efficiency.
